# Supplementary material for: Patterns of Intron Gain and Loss in Fungi
Source: PLoS Biol. 2004 Nov 30;2(12):e422. doi: 10.1371/journal.pbio.0020422 (PMC532390; doi:10.1371/journal.pbio.0020422)
Supplement: Table S1 — Also available at http://genes.mit.edu/NielsenEtAl/. (4.3 MB ZIP). [file pbio.0020422.st001.zip › NielsenEtAl/html/1388.html]

AN6366.1.NCU00216.1.MG06289.1.FG06947.1


```
 CLUSTAL W (1.82) Multiple Sequence Alignments - Introns Inserted


Sequence 1: NCU00216.1	310 aa
Sequence 2: FG06947.1	314 aa
Sequence 3: MG06289.1	309 aa
Sequence 4: AN6366.1	313 aa
Alignment Length: 320 aa
Number Identitical Residues: 170 aa
Alignment Score (without introns) 8199


MG06289.1 	MAP-----PLSSKVYVDGVYIPAALIVIGTAIVKRDWVVYSVALALALGTWKFFQ---LK
NCU00216.1	MS----EESLLERKYFDGIYIPAGLLVFGTVIVKKEWTLYAALAALILGAIKYFR---ML
FG06947.1 	MSSPADNDSLLAKHYIDYVYAPGLLLIVGTLIVKKEWTPYAALVAIAFGIYNFLA---FQ
AN6366.1  	MPVS----SALSLENITGVYAPSALLVVGTFILKKEWVPFAVALAAGFVAWKLSVGGSSK
          	*. .    .      .  :* *. *::.** *:*::*. ::.  *  :   :   ..:  

MG06289.1 	1PKKVLDPTKFQEFELKEKTIISHNVAI2YRIQLPSPSSILGLPIGQHISIGADIPQPDG
NCU00216.1	1PKKVLKPAVFQEFELKEKTIISHNVAI2YRFKLPTPDSVLGLPIGQHISIGAAIEQPDG
FG06947.1 	1VKKTLKPDVFQDFELEEKTIVSHNVAI2YRFKLPSPKHILGLPIGQHISIGAPCPQPDG
AN6366.1  	1PRKVLNPNEFQNFVLKEKNDISHNVTI~YRFALPRPTDILGLPIGQHISLAATIE---G
          	  :*.*.*  **:* *:**. :****:* **: ** *  :**********:.*      *

MG06289.1 	SSKEVVRSYTPISGDEQPGYVDLLIKSYPTGNISKYMAGLSVGQSIRVRGPKGAFVYQPN
NCU00216.1	STKEIVRSYTPISGDHQPGYFDLLIKSYPTGNISKHMASLQVGQTIRVKGPKGAFVYTPN
FG06947.1 	SVKEIVRSYTPISGDHQPGHVDLLIKSYPQGNISKHMASLVVGQTIKVRGPKGAFVYTPN
AN6366.1  	QPKEVVRSYTPISSDNEAGYFDLLVKAYPQGNISKYLTTLKVGDTMKVRGPKGAMVYTPN
          	. **:********.*.:.*:.***:*:** *****::: * **::::*:*****:** **

MG06289.1 	MVRHFGMIAGGTGITPMLQVVRAIVRGR---AAGDTTQVDLIFANVTKEDILLKEDLDAL
NCU00216.1	MVRHFGMVAGGTGITPMLQVIRAIVRGR---AAGDRTEVDLIFANVTAQDILLKEDLDAL
FG06947.1 	MVRHFGMIAGGTGITPMLQVIRAIVRGR---AAGDKTEVDLIFANVSPQDILLKEDLDAL
AN6366.1  	MCRHIGMIAGGTGITPMLQIIKAIIRNRPRNGGNDTTQVDLIFANVNPDDILLKDELEKL
          	* **:**:***********:::**:*.*. ....* *:********. :*****::*: *

MG06289.1 	AAEDKGFRVHYVLDRPPEGWTGGVGFVTQDMIT0KWLPKPADDVKILLCGPPPMVSGLKK
NCU00216.1	VAQDKGIRVHYVLDRPPEGWTGGVGFVTQEMVE~KLLPKPASDVKILLCGPPPMISGLKK
FG06947.1 	AAQDAGIRIHYVLDKPPEGWTGGVGYVTADMIN0KYLPKPADDVKILLCGPPPMISGLKK
AN6366.1  	AAEDDGFRIYYVLNNPPEGWTGGVGFVTPDMIK0ERLPAPASDIKILLCGPPPMVSAMKK
          	.*:* *:*::***:.**********:** :*:  : ** **.*:**********:*.:**

MG06289.1 	ATEALGFKKARPVSKLEDQVFAF
NCU00216.1	ATEALGFQKARPVSKLEDQVFAF
FG06947.1 	ATESLGFKKARPVSKLVDQVFAF
AN6366.1  	ATESLGYTKARPVSKLEDQVFCF
          	***:**: ******** ****.*
```
